# Waist-to-Height Ratio and Cardiovascular Risk Factors in Elderly Individuals at High Cardiovascular Risk

**DOI:** 10.1371/journal.pone.0043275

**Published:** 2012-08-14

**Authors:** Marta Guasch-Ferré, Mònica Bulló, Miguel Ángel Martínez-González, Dolores Corella, Ramon Estruch, María-Isabel Covas, Fernando Arós, Julia Wärnberg, Miquel Fiol, José Lapetra, Miguel Ángel Muñoz, Lluís Serra-Majem, Xavier Pintó, Nancy Babio, Andrés Díaz-López, Jordi Salas-Salvadó

**Affiliations:** 1 Human Nutrition Unit, Faculty of Medicine and Health Sciences, IISPV (Institut d’Investigació Sanitària Pere Virgili), Universitat Rovira i Virgili, Reus, Spain; 2 CIBERobn (Centro de Investigación Biomédica en Red Fisiopatología de la Obesidad y Nutrición), Institute of Health Carlos III, Madrid, Spain; 3 Department of Preventive Medicine and Public Health, University of Navarra, Pamplona, Spain; 4 Department of Preventive Medicine, University of Valencia, València, Spain; 5 Department of Internal Medicine, Hospital Clínic, University of Barcelona, Barcelona, Spain; 6 Cardiovascular Risk and Nutrition Research Group, Institut Municipal d’ Investigació Mèdica, Barcelona Biomedical Research Park, Barcelona, Spain; 7 Department of Cardiology, University Hospital Txagorritxu, Vitoria, Spain; 8 Department of Preventive Medicine, University of Malaga, Malaga, Spain; 9 Institute of Health Sciences, University of Balearic Islands and Hospital Son Espases, Palma de Mallorca, Spain; 10 Department of Family Medicine, Primary Care Division of Sevilla, San Pablo Health Center, Sevilla, Spain; 11 Primary Care Division, Catalan Institute of Health, Barcelona, Spain; 12 Department of Clinical Sciences, University of Las Palmas de Gran Canaria, Las Palmas, Spain; 13 Lipids and Vascular Risk Unit, Internal Medicine, Hospital Universitario de Bellvitge, Hospitalet de Llobregat, Barcelona, Spain; University of Sao Paulo, Brazil

## Abstract

**Introduction:**

Several anthropometric measurements have been associated with cardiovascular disease, type-2 diabetes mellitus and other cardiovascular risk conditions, such as hypertension or metabolic syndrome. Waist-to-height-ratio has been proposed as a useful tool for assessing abdominal obesity, correcting other measurements for the height of the individual. We compared the ability of several anthropometric measurements to predict the presence of type-2 diabetes, hyperglycemia, hypertension, atherogenic dyslipidemia or metabolic syndrome.

**Materials and Methods:**

In our cross-sectional analyses we included 7447 Spanish individuals at high cardiovascular risk, men aged 55–80 years and women aged 60–80 years, from the PREDIMED study. Logistic regression models were fitted to evaluate the odds ratio of presenting each cardiovascular risk factor according to various anthropometric measures. The areas under the receiver-operating characteristic curve (AUC) were used to compare the predictive ability of these measurements.

**Results:**

In this relatively homogeneous cohort with 48.6% of type-2 diabetic individuals, the great majority of the studied anthropometric parameters were significantly and positively associated with the cardiovascular risk factors. No association was found between BMI and body weight and diabetes mellitus. The AUCs for the waist-to-height ratio and waist circumference were significantly higher than the AUCs for BMI or weight for type-2 diabetes, hyperglycemia, atherogenic dyslipidemia and metabolic syndrome. Conversely, BMI was the strongest predictor of hypertension.

**Conclusions:**

We concluded that measures of abdominal obesity showed higher discriminative ability for diabetes mellitus, high fasting plasma glucose, atherogenic dyslipidemia and metabolic syndrome than BMI or weight in a large cohort of elderly Mediterranean individuals at high cardiovascular risk. No significant differences were found between the predictive abilities of waist-to-height ratio and waist circumference on the metabolic disease.

## Introduction

Overweight and obesity are major risk factors for type-2 diabetes mellitus, cardiovascular morbidity and mortality, cancer and other chronic health diseases and conditions [1], [2], [3]. To understand these associations, not only the total amount of body fat is important, but also the distribution of fat. Evidence suggests that central fat or “android distribution" is more strongly related to cardiovascular risk than peripheral fat or “gynoid distribution" [4]. This may be because abdominal obesity is associated with increased secretion of fatty acids, adipocytokines, hyperinsulinemia, insulin resistance, hypertension and atherogenic dyslipidemia, and leads to greater cardiovascular risk [5].

Several easily obtained anthropometric measurements, can predict diabetes and other risk conditions, such as hypertension or metabolic syndrome (MetS). Cross-sectional and prospective studies have shown that the waist-to-height-ratio (WHtR), waist circumference (WC), and body mass index (BMI) are able to predict diabetes and other cardiovascular conditions; however, WHtR and WC appeared to be better predictors than BMI, because of the relation between waist circumference and central obesity [6], [7], [8].

Although BMI is widely used to assess overweight and obesity, it does not distinguish fat from muscle or different fat distributions [9]. Other anthropometric measurements which have been proposed as useful tools for detecting obesity are the waist circumference or the waist-to-hip ratio. The main limitation of the waist-to-hip ratio is that both waist and hip can decrease with weight reduction and as a consequence changes in the ratio are frequently small. Waist circumference is also strongly associated with central obesity. Nevertheless, it may over- or under-evaluate risk for tall and short individuals with similar waist circumference [10].

In several studies conducted on Asian and Caucasian populations, WHtR outperformed BMI in the detection of impaired fasting plasma glucose (FPG), type-2 diabetes mellitus, hypertension, MetS and atherogenic dyslipidemia [8], [10]. However, with the exception of an Italian study in relatively few individuals [11]; and two studies in which they had included young and healthy adults from Crete [12] or Spain [13], this association has not been evaluated in Mediterranean populations. As far as we know, the predictive power of anthropometric measurements has not been assessed in elderly Mediterranean individuals at high cardiovascular risk, for whom the early detection of cardiovascular risk factors is essential if cardiovascular disease is to be prevented. The aim of the present study was to compare the predictive value of several anthropometric measurements on the presence of type-2 diabetes mellitus, hyperglycemia, hypertension, atherogenic dyslipidemia and metabolic syndrome in a large cohort of elderly Mediterranean individuals at high cardiovascular risk from the PREDIMED study.

## Materials and Methods

For PREDIMED Study the respective local institutional review boards approved the study protocol and all participants provided written informed consent.

The PREDIMED study (PREvención con DIeta MEDiterránea) is a large, parallel-group, multicenter, randomized, controlled clinical trial which aims to assess the effects of the Mediterranean diet on the primary prevention of cardiovascular disease (CVD) (www.predimed.org and www.predimed.es) (http://www.controlled-trials.com/ISRCTN35739639). The PREDIMED study was conducted in Spain. Recruitment took place between October 2003 and January 2009, and the 7447 participants were randomly assigned to one of three interventions (two Mediterranean diets enriched with extra virgin olive oil or mixed nuts, and a control low-fat diet). The design and methods used in the PREDIMED study have been described elsewhere [14].

Participants were men aged 55–80 years and women aged 60–80 years, who were free of CVD at baseline but who had either type-2 diabetes mellitus or fulfilled at least three or more coronary heart disease risk factors: current smoking, hypertension (blood pressure ≥140/90 mmHg or treatment with antihypertensive medication), high plasma LDL-cholesterol (≥160 mg/dL or lipid-lowering therapy), low plasma HDL-cholesterol (≤40 mg/dL in men and ≤50 mg/dL in women), overweight or obesity (BMI ≥25 kg/m^2^), and family history of premature CVD (≤55 years in men and ≤60 years in women). The exclusion criteria for the PREDIMED study were the presence of any severe chronic illness, previous history of CVD, alcohol or drug abuse, BMI ≥40 kg/m^2^ and history of allergy or intolerance to olive oil or nuts.

At baseline examination and yearly in follow-up visits, trained personnel performed anthropometric and blood pressure measurements and obtained samples of fasting blood. Weight and height were measured with light clothing and no shoes with calibrated scales and a wall-mounted stadiometer, respectively; waist circumference was measured midway between the lowest rib and the iliac crest using an anthropometric tape; blood pressure was measured using a validated oscillometer [Omron HEM705CP, Hoofddorp, Netherlands]. in triplicate with a 5-min interval between each measurement, and the mean of these values was recorded. We also administered a 137-item validated food frequency questionnaire [15]; the validated Spanish version of the Minnesota Leisure Time Physical Activity Questionnaire [16]; and a 47-item questionnaire about education, lifestyle, history of illnesses and medication use. Samples of serum, EDTA plasma, and urine were coded, shipped to central laboratories, and stored at −80°C until analysis. Laboratory analyses were performed on frozen serum samples. Serum glucose, cholesterol, and triglyceride concentrations were measured using standard enzymatic automated methods. HDL-cholesterol was measured by enzymatic procedure after precipitation.

For the present study, 7447 individuals were included in the statistical analysis to assess the association between anthropometric measurements and diabetes. For the other outcomes (hypertension, atherogenic dyslipidemia, metabolic syndrome or impaired fasting plasma glucose) fewer number of individuals (ranging between 3335 and 7447) were included in the analyses because the biochemical parameters from all participants were not available.

The criteria for cardiovascular risk factors analysed were the following: Type-2 diabetes mellitus was defined as previous diagnosis done by a physician using the WHO criteria [17]; hyperglycemia (defined as having FPG ≥110 mg/dL ) only non diabetic individuals at baseline were included [18]; hypertension (defined as systolic blood pressure (SBP) ≥130 mmHg, diastolic blood pressure (DBP) ≥85 mmHg or if subjects were receiving antihypertensive treatment); atherogenic dyslipidemia, which was defined as HDL-cholesterol <40 mg/dL (<0.9 mmol/L) in men or <50 mg/dL (<1.2 mmol/L) in women and TG was ≥150 mg/dL (≥1.7 mmol/L). Metabolic syndrome and its components were defined by the updated harmonized International Diabetes Federation (IDF) and the American Heart, Lung, and Blood Institute (AHA/NHLBI) criteria [19]. MetS was considered to be present when subjects fulfilled three or more of the following criteria: a) abdominal obesity (defined as waist circumference ≥102 cm in men or ≥88 cm in women), b) low HDL cholesterol (defined as <40 mg/dL in men or <50 mg/dL in women or if subjects were receiving fibrate treatment), c) hypertriglyceridemia (defined as triglyceride concentrations ≥150 mg/dL or fibrate treatment), d) hypertension (defined as a blood pressure level of ≥130/85 mmHg or if subjects were receiving antihypertensive medication), and e) hyperglycemia (defined as FPG concentration ≥100 mg/dL (≥5.6 mmol/L) or drug treatment for elevated glucose).

Because no interactions were observed between sex and the main outcomes, analyses were conducted for men and women together. To determine which anthropometric index was the best predictor of the metabolic risk factors, two statistical methods were used. First, multivariable logistic regression analysis was used to evaluate the association between baseline anthropometric measurements (WHtR, calculated as waist in cm divided by height in cm; waist circumference; BMI, calculated as weight in kg divided by height in m^2^; and body weight) and cardiovascular risk conditions. The adjusted odds ratios (ORs) and 95% confidence intervals (95%CI) were presented per one standard deviation change in the respective anthropometric parameter. Four models were fitted for each measurement: the unadjusted model; model 1, adjusted for sex, age (continuous) and region; model 2, adjusted for sex, age, region, current smoking (yes or no), education (illiterate, elementary education, secondary education, university degree) and marital status (single, widow, separated, religious, married); model 3 or fully-adjusted model, adjusted for sex, age, region, current smoking (yes or no), education (illiterate, elementary education, secondary education, university degree) and marital status (single, widow, separated, religious, married), total energy intake (continuous variable) and physical activity (continuous). Secondly, we calculated the areas under the receiver-operating characteristic (ROC) curves (AUC) for each model.

The level of significance for all statistical tests was set at P<0.05 for bilateral contrasts. Data were analyzed using the SPSS statistical package version 19.0. We used Epidat 3.1 software to estimate the statistical significance of the differences between the ROC curves areas of all the models according to each cardiovascular risk factor and according to the algorithm developed by DeLong and colleagues [20].

## Results

The baseline characteristics of anthropometric measurements and biochemical parameters are presented in Table 1. The mean age of the population was 67 (standard deviation ±6 years). Of all the subjects analyzed, 82.7% had hypertension, 48.6% were diabetic, 13.2% had atherogenic dyslipidemia, and 63.7% metabolic syndrome.

**Table 1:** Baseline characteristics of the study participants.

			TOTAL
n			7447
**Age, years**			67±6
**Weight, kg**			76.78±11.94
**Body mass index, kg/m^2^**			29.96±3.85
**Waist circumference, cm**			100.45±10.34
**Current smoker, n (%)**			1047 (14.1)
**Physical activity (METS-h/d)**			3.84±3.98
**Systolic blood pressure, mmHg**			148.61±20.07
**Diastolic blood pressure, mmHg**			82.84±10.58
**Hypertension, n (%)**			6162 (82.7)
**Diabetes, n (%)**			3616 (48.6)
**Atherogenic dyslipidemia, n (%)**			898 (13.2)
**Metabolic syndrome, n (%)**			4399 (63.7)
**Hypercholesterolemia, n (%)**			5383 (72.3)
**Total cholesterol, mg/dL**			206.76±38.11
**HDL cholesterol, mg/dL**			52.77±13.16
**LDL cholesterol, mg/dL**			130.31±33.98
**Triglycerides, mg/dL**			133.54±74.85
**Fasting plasma glucose, mg/dL**			120.56±41.11
**Marital status**	**Single, n (%)**		302 (4.1)
	**Married, n (%)**		5673 (76.3)
	**Widowed, n (%)**		1219 (16.4)
	**Divorced, n (%)**		221 (3.0)
**Educational level Primary education or** **less, n (%)**			5792 (77.8)
	**Secondary education, n (%)**		1121 (15.1)
	**Higher education, n (%)**		534 (7.2)
**Use of antihypertensive agents, % (n)**			5416 (72.7)
**Use of oral hypoglycaemic agents, % (n)**			2386 (32.0)

Abbreviations: HDL, high-density lipoprotein; LDL, low-density lipoprotein. Data are expressed as mean ± SD or number (percent).

Table 2 summarizes the odds ratios (ORs) and the AUCs for the associations between baseline anthropometric measurements and the prevalence of hyperglycemia and diabetes. When we analyzed the prevalence of hyperglycemia in non diabetic individuals, including 3335 individuals in the analysis, the ORs for each additional standard deviation increase were higher for WHtR (OR: 1.42; 1.29–1.56) and WC (OR: 1.43; 1.29–1.58) followed by BMI (OR: 1.39; 1.26–1.53) and body weight (OR: 1.37; 1.23–1.52) in the fully-adjusted model. In all models, the inclusion of waist circumference was associated with a larger AUC than the inclusion of weight (P<0.05); The AUC was also larger for models containing WHtR than those containing weight in models 1, 2 and in the fully-adjusted model (P<0.05). These results were similar when we included in the analysis both diabetic and non-diabetic individuals, or when the cut-off point for FPG was set at ≥100 mg/dL (data not shown). In the models evaluating the presence of diabetes, 7447 individuals were included; we observed that WHtR and waist circumference were positively associated with the prevalence of diabetes, whereas these associations were not apparent for BMI or for body weight. In the unadjusted models the AUCs when using WHtR and WC as measurements of adiposity were significantly greater than the AUC when weight was used instead. In the model 1 and 2, the AUCs of models including WHtR and WC outperformed those including BMI (P<0.05). In the fully-adjusted model the AUC of model including WHtR also outperformed those including BMI (P<0.05).

**Table 2:** Association between baseline anthropometric measurements (OR per one additional standard deviation) and the prevalence of high FPG of non diabetic individuals at baseline or T2DM. ROC analysis with Areas Under the Curve (AUC).

	WHtR			Waist Circumference			BMI		Weight			
	OR (95% CI)	AUC (95% CI)		OR (95% CI)	AUC (95% CI)		OR (95% CI)	AUC (95% CI)	OR (95% CI)		AUC (95% CI)	
**FPG ≥110 mg/dL (n = 3335)**												
**Unadjusted**	1.37 (1.25–1.51)	0.59^b^ (0.57–0.62)	1.47 (1.34–1.62)			0.61 (0.58–0.63)	1.33 (1.22–1.46)	0.59^b^(0.56–0.61)	1.39 (1.27–1.52)		0.59^b^(0.56–0.62)	
**Model 1**	1.42 (1.30–1.57)	0.61 (0.59–0.64)	1.43 (1.29–1.58)			0.61 (0.59–0.64)	1.39 (1.26–1.52)	0.61 (0.58–0.63)	1.36 (1.23–1.51)		0.59a,b(0.57–0.62)	
**Model 2**	1.42 (1.29–1.56)	0.62 (0.59–0.64)	1.42 (1.29–1.57)			0.62 (0.59–0.64)	1.38 (1.26–1.52)	0.61 (0.58–0.64)	1.36 (1.23–1.51)		0.60a,b(0.57–0.63)	
**Model 3**	1.42 (1.29–1.56)	0.62 (0.60–0.65)	1.43 (1.29–1.58)			0.62 (0.59–0.65)	1.39 (1.26–1.53)	0.61 (0.59–0.64)	1.37 (1.23–1.52)		0.60a,b(0.67–0.63)	
**DIABETES MELLITUS (n = 7447)**												
**Unadjusted**	1.12 (1.07–1.17)	0.53^b^ (0.52–0.54)		1.16 (1.11–1.21)	0.54 (0.52–0.55)		0.96 (0.92–1.01)	0.51^b^ (0.49–0.52)		1.01 (0.97–1.06)		0.50a,b(0.49–0.52)
**Model 1**	1.10 (1.05–1.15)	0.58 (0.56–0.59)		1.12 (1.06–1.17)	0.57^a^ (0.56–0.59)		0.99 (0.94–1.04)	0.57a,b(0.55–0.58)		0.95 (0.91–1.01)		0.57^a^(0.56–0.58)
**Model 2**	1.09 (1.04–1.14)	0.59 (0.58–0.61)		1.11 (1.05–1.16)	0.59 (0.58–0.60)		0.97 (0.93–1.02)	0.58a,b(0.57–0.60)		0.95 (0.90–1.01)		0.59 (0.57–0.60)
**Model 3**	1.12 (1.07–1.18)	0.61 (0.60–0.63)		1.11 (1.06–1.17)	0.61 (0.60–0.62)		0.98 (0.93–1.02)	0.61^a^ (0.59–0.62)		0.96 (0.91–1.01)		0.61 (0.60–0.62)

Regression logistic models: Odds Ratio for one SD increase in anthropometric measurements. Model 1, adjusted for sex, age (continuous) and region; Model 2, adjusted for sex, age, region, current smoking (yes or no), education (illiterate, elementary education, secondary education, university degree) and marital status (single, widow, separated, religious, married); Model 3, adjusted for sex, age, region, current smoking (yes or no), education (illiterate, elementary education, secondary education, university degree), marital status (single, widow, separated, religious, married), total energy intake (continuous), physical activity (continuous). Abbreviations: FPG, fasting plasma glucose; T2DM, type-2 diabetes mellitus; WHtR, waist-to-height ratio; BMI, body mass index.

a P value <0.05 compared to WHtR.

b P value <0.05 compared to waist circumference.

Table 3 shows the ORs and the AUCs for the associations between anthropometric measurements and the prevalence of various cardiovascular risk factors. All the anthropometric parameters were positively associated with these cardiovascular risk factors. We included 7414 individuals in the models evaluating hypertension. BMI appeared to be the best predictor of hypertension. In the first model, the AUC for BMI was significantly greater than the AUC for WHtR or waist circumference but the magnitude of the difference was tiny (AUCs: 0.67; 0.66; 0.66, respectively); also in the fully-adjusted model the AUC for the prediction using BMI (AUC: 0.68) was significantly better than that using WHtR or waist circumference (AUCs: 0.67; P<0.05 for the comparison between both areas). A total of 6786 individuals were included in the evaluation of atherogenic dyslipidemia. WHtR and WC had higher ORs than BMI or body weight. These measurements showed a reasonably good predictive value; in the fully-adjusted model, the AUCs for models including WHtR or WC (AUC: 0.62 for both) were significantly greater than for those including weight (AUC: 0.60; P<0.05).

**Table 3:** Association between baseline anthropometric measurements (OR per one additional standard deviation) and the prevalence of cardiovascular risk factors. ROC analysis with Areas Under the Curve (AUC).

	WHtR		Waist Circumference					BMI				Weight			
	OR (95% CI)	AUC (95% CI)	OR (95% CI)		AUC (95% CI)			OR (95% CI)		AUC (95% CI)		OR (95% CI)		AUC (95% CI)	
**HYPERTENSION (n = 7414)**															
**Unadjusted**	1.64 (1.48–1.82)	0.63 (0.60–0.66)	1.53 (1.39–1.69)		0.61 (0.59–0.64)			1.69 (1.52–1.89)	0.63 (0.61–0.66)			1.42 (1.28–1.58)		0.59^a^(0.57–0.62)	
**Model 1**	1.57 (1.42–1.75)	0.66 (0.63–0.69)	1.59 (1.43–1.77)		0.66 (0.63–0.69)			1.73 (1.55–1.93)	0.67a,b(0.65–0.70)			1.72 (1.53–1.94)		0.67 (0.64–0.69)	
**Model 2**	1.57 (1.41–1.74)	0.66 (0.64–0.69)	1.59 (1.43–1.77)		0.67 (0.64–0.69)			1.71 (1.53–1.91)	0.68a,b (0.65–0.71)			1.71 (1.52–1.93)		0.67 (0.65–0.70)	
**Model 3**	1.58 (1.43–1.76)	0.67 (0.64–0.70)	1.60 (1.44–1.78)		0.67 (0.64–0.70)			1.73 (1.55–1.94)	0.68a,b(0.66–0.71)			1.73 (1.53–1.95)		0.68 (0.66–0.71)	
**ATHEROGENIC DYSLIPIDEMIA (n = 6786)**															
**Unadjusted**	1.39 (1.29–1.49)	0.60 (0.58–0.62)		1.29 (1.20–1.38)			0.57^a^(0.55–0.59)	1.36 (1.27–1.46)			0.59^b^(0.57–0.61)		1.16 (1.09–1.25)		0.54a,b(0.52–0.56)
**Model 1**	1.36 (1.27–1.46)	0.61 (0.59–0.63)		1.36 (1.27–1.46)			0.61 (0.59–0.63)	1.32 (1.24–1.42)			0.60 (0.59–0.62)		1.32 (1.22–1.42)		0.59a,b(0.58–0.62)
**Model 2**	1.37 (1.28–1.47)	0.62 (0.60–0.64)		1.37 (1.28–1.47)			0.62 (0.60–0.64)	1.34 (1.25–1.43)			0.61 (0.59–0.63)		1.31 (1.22–1.43)		0.60a,b(0.58–0.62)
**Model 3**	1.36 (1.27–1.46)	0.62 (0.60–0.64)		1.36 (1.27–1.46)			0.62 (0.60–0.64)	1.32 (1.23–1.42)			0.61 (0.60–0.63)		1.31 (1.21–1.41)		0.60a,b(0.59–0.62)
**METABOLIC SYNDROME (n = 6906)**															
**Unadjusted**	2.74 (2.51–2.93)	0.74 (0.72–0.75)		2.47 (2.32–2.63)		0.72^a^(0.71–0.73)		2.14 (2.01–2.27)			0.69a,b(0.68–0.70)		1.67 (1.58–1.76)	0.63a,b(0.62–0.65)	
**Model 1**	2.73 (2.55–2.92)	0.74^b^(0.73–0.75)		2.98 (2.78–3.19)		0.76 (0.74–0.77)		2.12 (1.99–2.25)			0.70a,b(0.68–0.71)		2.20 (2.06–2.35)	0.69a,b(0.68–0.70)	
**Model 2**	2.73 (2.55–2.92)	0.74^b^(0.73–0.75)		2.97 (2.77–3.19)		0.76 (0.74–0.77)		2.11 (1.98–2.24)			0.70a,b(0.68–0.71)		2.21 (2.07–2.36)	0.69a,b(0.68–0.71)	
**Model 3**	2.70 (2.52–2.89)	0.74^b^ (0.73–0.76)		2.95 (2.75–3.17)		0.76 (0.75–0.77)		2.09 (1.97–2.23)			0.70a,b(0.69–0.72)		2.20 (2.06–2.35)	0.70a,b(0.69–0.72)	

Regression logistic models: Odds Ratio for one SD increase in anthropometric measurements. Model 1, adjusted by sex, age (continuous) and region; Model 2, adjusted by sex, age, region, current smoking (yes or no), education (illiterate, elementary education, secondary education, university degree) and marital status (single, widow, separated, religious, married); Model 3, adjusted for sex, age, region, current smoking (yes or no), education (illiterate, elementary education, secondary education, university degree), marital status (single, widow, separated, religious, married), total energy intake (continuous), physical activity (continuous). Abbreviations: WHtR, waist-to-height ratio; BMI, body mass index.

a P value <0.05 compared to WHtR.

b P value <0.05 compared to waist circumference.

The strongest associations were observed for metabolic syndrome, which was evaluated in 6906 individuals (Table 3). For one SD increase in WHtR the odds of having MetS was 2.70 (95% CI: 2.52–2.89) and the OR of WC was 2.95 (2.75–3.17) in the fully-adjusted model. These measures also had a good predictive ability; their inclusion in the fully-adjusted models (model 3) showed AUCs of 0.74 for WHtR and 0.76 for WC. Moreover, in all models the AUCs after inclusion of either WHtR or WC significantly outperformed the areas under the curve when BMI or body weight were used instead (P<0.05).

## Discussion

In the present study conducted in a large Mediterranean cohort at high risk of CVD, our main finding was that measures of overall and central obesity were strongly associated not only with the prevalence of hyperglycemia and diabetes, but also with other cardiovascular risk factors, such as hypertension, atherogenic dyslipidemia and metabolic syndrome. These risk factors were used in our analysis because they have been associated with obesity and are independent predictors of cardiovascular disease [3].

Our study shows that individuals with greater adiposity indices were at greater risk of having diabetes and other cardiovascular risk factors. The ROC analysis proved that measures of central obesity (WHtR and WC) showed higher discriminative ability for FPG, diabetes, atherogenic dyslipidemia and MetS than BMI or body weight, but not for hypertension. Contrary to our expectations, no significant differences were found between the predictive abilities of WHtR and WC.

The general findings of the present study are in agreement with those of previous studies which showed that the AUC for WHtR was similar to WC at predicting diabetes and related risk factors [21], [22]. A systematic review published by Browning et al., which analysed the predictive value of several adiposity indices for CVD and metabolic conditions, showed that in 86% of studies in men and 91% in women the AUC of WHtR was higher than or equal to WC [10]. This could be explained because both measurements reflect central adiposity and WHtR was calculated using WC.

Some authors have suggested correcting waist circumference for the height of the individual, so the waist-to-height-ratio has been proposed as a useful tool for assessing abdominal obesity [6], [22], [23]. In addition, as height has been inversely associated with cardiovascular diseases (CVDs), it may be important to correct waist circumference for height [24]. The importance of this point is further highlighted if we take into account that height remains quite unchanged during adulthood, so WHtR will change only when there is a change in the waist measurement, whereas other indices, such as waist-to-hip ratio are more sensitive to changes in body size, and both hip and waist could increase or decrease proportionately. Furthermore, the meta-analyses that have been carried out support the evidence that measures of centralized obesity, especially WHtR, are better at detecting cardiovascular risk factors in both men and women than BMI or other anthropometric measurements [7], [8].

In our study, BMI appeared to be the anthropometric measurement which better discriminates the prevalence of hypertension. This result is consistent with a previous study of 3006 Chinese adults in which, the BMI OR for hypertension was higher than measures of central obesity in men [25]. Furthermore, although the great majority of studies assessing anthropometric measurements and the prevalence of diabetes have shown a positive association between BMI and diabetes, our study has not; in just the same way that it was not found in a cross-sectional population-based study conducted in 5073 Iranian women [26]. Hadaegh et al. also showed no significant risk across BMI and incident diabetes in a prospective cohort of men [27].

In line with other studies, our results suggest that measures of central obesity (WHtR and WC) are more associated with MetS than other anthropometric measurements. The AUCs of WHtR and WC were significantly higher than the AUCs of BMI and body weight, and the predictive capability of these measures for MetS is quite good (higher than 70%). Previous studies also found that WHtR may be the most effective anthropometric index for screening MetS in different populations [11], [28].

Several studies have examined the association between anthropometric measurements and hypertriglyceridemia or hypercholesterolemia [22], [29]; even though we found that no studies assessed the association of these measurements with atherogenic dyslipidemia prevalence. In our population, measures of central obesity also showed higher discriminative ability for atherogenic dyslipidemia than body weight.

The main limitation of the present study was the use of cross-sectional data to assess the ability of anthropometric measurements to predict CVD risk factors. Further longitudinal analyses will provide stronger evidence of these associations. Another limitation was that the analyses were conducted in an elderly population at high risk for CVD, these individuals were included in the PREDIMED study for having either type-2 diabetes or other CVD risk factors, so they do not represent the usual variability that we could find in the general population, and results may not be extrapolated to the general population. It should be kept in mind the potentiality for reverse causality bias. It is highly plausible that individuals who have several cardiovascular risk factors or are obese receive more advice from their physicians or dieticians than general population, so they would be more likely to improve their lifestyle and their dietary habits. However, the sample of individuals studied was larger than that of most previous studies and they were all subjects at high cardiovascular risk. This strongly reinforces the interest of our results. Moreover, no previous studies have been conducted in elderly Mediterranean individuals at high cardiovascular risk, so our results extend knowledge on the association between adiposity and disease in this particular population where the earlier detection of cardiovascular risk factors is essential if CVD is to be prevented.

In conclusion, measures of abdominal obesity (WHtR or WC) showed higher discriminative ability for diabetes mellitus, high fasting plasma glucose, atherogenic dyslipidemia and metabolic syndrome than BMI or body weight in a large cohort of elderly Mediterranean individuals at high cardiovascular risk.
